# Hot Speech and Exploding Bombs: Autonomic Arousal During Emotion Classification of Prosodic Utterances and Affective Sounds

**DOI:** 10.3389/fpsyg.2018.00228

**Published:** 2018-02-28

**Authors:** Rebecca Jürgens, Julia Fischer, Annekathrin Schacht

**Affiliations:** ^1^Cognitive Ethology Laboratory, German Primate Center, Göttingen, Germany; ^2^Department of Affective Neuroscience and Psychophysiology, Institute of Psychology, University of Göttingen, Göttingen, Germany; ^3^Leibniz ScienceCampus Primate Cognition, Göttingen, Germany

**Keywords:** vocal emotion expressions, autonomic responses, skin conductance, pupillometry, emotion, arousal

## Abstract

Emotional expressions provide strong signals in social interactions and can function as emotion inducers in a perceiver. Although speech provides one of the most important channels for human communication, its physiological correlates, such as activations of the autonomous nervous system (ANS) while listening to spoken utterances, have received far less attention than in other domains of emotion processing. Our study aimed at filling this gap by investigating autonomic activation in response to spoken utterances that were embedded into larger semantic contexts. Emotional salience was manipulated by providing information on alleged speaker similarity. We compared these autonomic responses to activations triggered by affective sounds, such as exploding bombs, and applause. These sounds had been rated and validated as being either positive, negative, or neutral. As physiological markers of ANS activity, we recorded skin conductance responses (SCRs) and changes of pupil size while participants classified both prosodic and sound stimuli according to their hedonic valence. As expected, affective sounds elicited increased arousal in the receiver, as reflected in increased SCR and pupil size. In contrast, SCRs to angry and joyful prosodic expressions did not differ from responses to neutral ones. Pupil size, however, was modulated by affective prosodic utterances, with increased dilations for angry and joyful compared to neutral prosody, although the similarity manipulation had no effect. These results indicate that cues provided by emotional prosody in spoken semantically neutral utterances might be too subtle to trigger SCR, although variation in pupil size indicated the salience of stimulus variation. Our findings further demonstrate a functional dissociation between pupil dilation and skin conductance that presumably origins from their differential innervation.

## Introduction

Emotional expressions conveyed by face, voice and in body gestures are strong social signals and might serve as emotion-elicitors in a spectator or listener. Situations that are of relevance for someone’s wellbeing or future prospects, such as meeting an aggressor on the street, possess an emotional meaning that has the power to trigger emotions in the beholder. Bodily reactions, one of the key components of emotion (Moors et al., 2013), are regulated by the autonomous nervous system (ANS), and include changes in the cardiovascular system, in respiration and perspiration (Kreibig, 2010). While autonomic responses to affective pictures and sounds have been reliably demonstrated (e.g., Bradley et al., 2001a), only little is known about ANS responses to emotional expressions, in particular with regard to spoken language. Emotional expressions in the voice, however, are of special relevance considering that speech might be the most important communication channel in humans. Our study therefore had two main aims; first, we investigated autonomic activation in response to spoken utterances of neutral semantic content but varying in their emotional prosody, and second, we compared these responses to those triggered by another auditory domain, namely affective sounds.

There are various physiological indicators reflecting autonomic responses during emotion processing. Skin conductance responses (SCRs) are one of the most frequently used peripheral physiological markers; presumably because they are exclusively activated by the sympathetic nervous system and because they are robust against voluntary modulations. Thus, they can be assumed to provide an excellent measure for the elicitation of emotional arousal (Dawson et al., 2007). Another promising indicator of even unconscious and subtle changes of emotional arousal are changes of the pupil size during stimulus processing (Laeng et al., 2012). The size of the pupil diameter is controlled by two muscles, innervated by both sympathetic and parasympathetic branches of the ANS that receive input from parts of the central nervous system involved in cognitive and affective processing (e.g., Hoeks and Ellenbroel, 1993). A vast body of research has suggested that pupillary responses serve as a potent measure for top-down and bottom-up attention (e.g., Laeng et al., 2012; Riese et al., 2014), both with regard to emotional and motivational processing (e.g., Bayer et al., 2010, 2017a,b; Bradley et al., 2008; Partala and Surakka, 2003; Võ et al., 2008) and cognitive load (e.g., Stanners et al., 1979; Verney et al., 2001; Nuthmann and Van der Meer, 2005; Van der Meer et al., 2010). An increased attention or mental effort is accompanied by enlarged pupil dilations: the more attention, the larger the pupil size. During emotion perception and emotion recognition, pupil dilation can be influenced by both emotion-based and cognitive factors. The simultaneous consideration of SCRs and changes of the pupil size might therefore help to separate the emotion-related from the cognitive sub-processes during processing of emotional information.

Affective pictures or sounds, mainly representing violence and erotica, have been shown to robustly increase SCRs and pupil dilations of the perceiver (Partala and Surakka, 2003; Bradley et al., 2008; Lithari et al., 2010). While the processing of emotional expressions has been shown to evoke emotion-related pupil size changes (see Kuchinke et al., 2011 for prosodic stimuli, Laeng et al., 2013 for faces), evidence for increased SCRs to emotional expressions is, however, less clear (Alpers et al., 2011; Aue et al., 2011; Wangelin et al., 2012). Alpers et al. (2011) and Wangelin et al. (2012) directly compared SCRs to emotional faces and affective scenes. Both studies found increased SCRs to arousing scenes compared to neutral ones, but not in response to facial expressions of emotion. In contrast, Merckelbach et al. (1989) reported stronger SCRs to angry compared to happy faces, while Dimberg (1982) did not find any differences between the two conditions. SCRs to emotional prosody have been even less investigated: Aue et al. (2011) studied the influence of attention and laterality during processing of angry prosody. Compared to neutrally spoken non-sense words, the angry speech tokens caused higher SCRs. In line with this finding, Ramachandra et al. (2009) demonstrated that nasals pronounced in an angry or fearful tone of voice elicit larger SCRs in the listener than neutrally pronounced ones, but their stimulus set only consisted of an extremely limited number of stimuli. A direct comparison between ANS responses to prosodic utterances vs. affective sounds, both conveying emotional stimuli of the same modality, has not been conducted so far.

The inconsistencies in the studies mentioned above might be explained by the absence of contexts, in which the stimuli were presented to the participants. Experimental setups conducted with entirely context-free presentation of emotional expressions, which are unfamiliar and also unimportant for the participants may simply reduce the overall social relevance of these stimuli and therefore fail to trigger robust emotion-related bodily reactions. In a recent study, Bayer et al. (2017b) demonstrated the importance of context. The authors observed increased pupil dilations to sentence-embedded, written words of emotion content in semantic contexts of high individual relevance. Similarly, perceived similarity to a person in distress increases emotional arousal in a bystander (Cwir et al., 2011). In general, sharing attitudes, interests, and personal characteristics with another person have been shown to immediately create a social link to that person (Vandenbergh, 1972; Miller et al., 1998; Jones et al., 2004; Walton et al., 2012). We therefore intended to vary the relevance of speech stimuli by embedding them into context and manipulating the idiosyncratic similarity between the fictitious speakers and participants.

The first aim of the present study was to test whether spoken utterances of varying emotional prosody trigger arousal-related autonomic responses, measured by pupil dilation and skin conductance in an explicit emotion categorization task. We increased the social relevance of our speech samples by providing context information with manipulated personal similarity in terms of biographical data between the participant and a fictitious speaker. Second, we examined participants’ physiological responses to affective sounds in comparison to the prosodic utterance. These affective sounds were for instance exploding bombs, or applause. Based on previous findings on emotional stimuli in the visual modality, we predicted stronger arousal-related effects for the affective sounds than for prosodic stimuli. Finally, we implemented a speeded reaction time task on the prosodic and sound stimuli in order to disentangle the cognitive and emotion-based modulations of the two physiological markers, by examining the cognitive difficulties during explicit recognition of the prosodic utterances and affective sounds.

## Materials and Methods

### Ethics Statement

The present study was approved by the local ethics committee of the Institute of Psychology at the Georg-August-Universität Göttingen. All participants were fully informed about the procedure and gave written informed consent prior to the experiment.

### Participants

Twenty-eight female German native speakers, ranging in age between 18 and 29 years (*M* = 22.8), participated in the main study. The majority of participants (23 out of 28) were undergraduates at the University of Göttingen, three just finished their studies and two worked in a non-academic profession. Due to technical problems during recordings, two participants had to be excluded from analyses of pupil data. We restricted the sample to female participants in order to avoid sex-related variability in emotion reactivity (Bradley et al., 2001b; Kret and De Gelder, 2012).

### Stimuli

#### Spoken Utterances With Emotional Prosody

The emotional voice samples were selected from the Berlin Database of Emotional Speech (EmoDB, Burkhardt et al., 2005). The database consists of 500 acted emotional speech tokens of 10 different sentences. These sentences were of neutral meaning, such as “The cloth is lying on the fridge” [German original “Der Lappen liegt auf dem Eisschrank”], or “Tonight I could tell him” [“Heute abend könnte ich es ihm sagen”]. From this database we selected 30 angry, 30 joyful, and 30 neutral utterances, spoken by five female actors. Each speaker provided 18 stimuli to the final set (6 per emotion category). The stimuli had a mean duration of 2.48 ± 0.71 s (anger = 2.61 ± 0.7, joy = 2.51 ± 0.71, and neutral = 2.32 ± 0.71), with no differences between the emotion categories (Kruskal–Wallis chi-squared = 2.893, *df* = 2, *p* = 0.24). Information about the recognition of indented emotion and perceived naturalness were provided by Burkhardt et al. (2005). We only chose stimuli that were recognized well above chance and perceived as convincing and natural (Burkhardt et al., 2005). Recognition rates did not differ between emotion categories (see **Table 1** for descriptive statistics, Kruskal–Wallis chi-squared = 5.0771, *df* = 2, *p* = 0.079). Anger stimuli were, however, perceived as more convincing than joyful stimuli (Kruskal–Wallis chi-squared = 11.1963, *df* = 2, *p* = 0.004; *post hoc* test with Bonferroni adjustment for anger – joy *p* = 0.003). During the experiment, we presented prosodic stimuli preceded by short context sentences that were presented in written form on the computer screen. With this manipulation we aimed at providing context information in order to increase the plausibility of the speech tokens. These context sentences were semantically related to the prosodic target sentence and neutral in their wording, such as “She points into the kitchen and says” [German original: “Sie deutet in die Küche und sagt”] followed by the speech token “The cloth is laying on the fridge” [“Der Lappen liegt auf dem Eisschrank”] or “She looks at her watch and says” [German original “Sie blickt auf die Uhr und sagt”] followed by the speech “It will happen in 7 h” [“In sieben Stunden wird es soweit sein”].

**Table 1:** Descriptive statistic of the stimulus material.

Prosodic stimuli^a^	% Recognition (Mean ±*SD*)	% Naturalness (Mean ±*SD*)
Anger	96.17 ± 7.39	84.55 ± 10.61
Neutral	93.52 ± 6.46	80.08 ± 11.16
Joy	93.19 ± 8.85	72.51 ± 15.98

**Sounds^b^**	**Pleasantness (Mean ± *SD*)**	**Arousal (Mean ± *SD*)**

Negative arousing	2.8 ± 1.76	6.9 ± 1.86
Neutral	4.91 ± 1.75	4.46 ± 2.04
Positive arousing	7.23 ± 1.78	6.75 ± 1.81

^a^*Burkhardt et al., 2005;*^b^*Bradley and Lang, 2007. Given are the percentage of correct recognition and the percentage of perceived naturalness of the selected prosodic stimuli (Data based on Burkhardt et al., 2005). Sounds were rated on a 1–9 likert scale (1-negative, 9-positiv/1-not aroused, 9-aroused) by Bradley and Lang (2007). Given is the mean and SD for the selected sample.*

#### Affective Sounds

Forty-five affective sounds (15 arousing positive, 15 arousing negative, 15 neutral^1^) were selected from the IADS database (International Affective Digital Sounds, Bradley and Lang, 1999). All of them had a duration of 6 s. Erotica were not used in our study as they have been shown to be processed differently compared to other positive arousing stimuli (Partala and Surakka, 2003; van Lankveld and Smulders, 2008). The selected positive and negative stimuli did not differ in terms of arousal (see **Table 1** for descriptive statistics; *t*(27) = -0.743, *p* = 0.463) and were significantly more arousing than the neutral stimuli (*t*(25) = 12.84, *p* < 0.001). In terms of emotional valence, positive and negative stimuli differed both from each other (*t*(24) = 21.08, *p* < 0.001) and from the neutral condition (positive–neutral *t*(19) = 11.99, *p* < 0.001, negative–neutral *t*(25) = 15,15, *p* < 0.001), according to the ratings provided in the IADS database. Positive and negative sounds were controlled for their absolute valence value from the neutral condition (*t*(24) = 0.159, *p* = 0.875). Note that this stimulus selection was based on ratings by female participants’ ratings only, provided by Bradley and Lang (2007).

As the emotional sounds were rather diverse in their content, we controlled for differences in specific acoustic parameters that might trigger startle reactions or aversion and thus influence the physiological indicators used in the present study in an unintended way. These parameters included intensity, intensity onset (comprising only the first 200 ms), intensity variability (intensity standard deviation), noisiness, harmonic-to-noise ratio (HNR) and energy distribution (frequency at which 50% of energy distribution in the spectrum was reached). Intensity parameters were calculated using Praat (Boersma and Weenink, 2009), while noisiness, energy distribution and HNR were obtained by using LMA (Lautmusteranalyse developed by K. Hammerschmidt – Schrader and Hammerschmidt, 1997; Hammerschmidt and Jürgens, 2007; Fischer et al., 2013). We calculated linear mixed models in R to compare these parameters across the three emotion categories (see **Table 2**). We conducted *post hoc* analysis even when the general analysis was only significant at trend level. We found differences at trend level for intensity and intensity variability, and significant effects for energy distribution across the emotion categories. Differences were marginal and unsystematically spread across the categories, meaning that no emotion category accumulates all aversion related characteristics (see **Table 2**). Differences rather depict the normal variation when looking at complex sounds. The probability that acoustic structure confounds the physiological measure is thus low.

**Table 2:** Acoustic parameter values for the affective sounds grouped by valence.

Parameter	Negative (Mean ±*SD*)	Neutral (Mean ±*SD*)	Positive (Mean ±*SD*)
Intensity [db]	65.66 ± 13.07	64.57 ± 9.33^a^	71.50 ± 6.62^a^
Intensity onset [db]	61.28 ± 18.64	62.44 ± 9.04	68.36 ± 7.20
Intensity variability [db]	12.66 ± 7.66^a^	8.48 ± 4.07	7.83 ± 7.64^a^
% noise	68.07 ± 34.13	80.33 ± 24.94	58.73 ± 33.03
harmonic-to-noise ratio	390.50 ± 469.72	369.80 ± 235.05	665.09 ± 638.37
50% Energy distribution [Hz]	1802.40 ± 961.37^b^	1046.67 ± 868.05^b^	1231.67 ± 389.62

*Differences in one parameter across emotion are indicated by uncapitalized letters.*^a^*p < 0.1, ^*b*^p < 0.05.*

### Similarity Manipulation

On the basis of participants’ demographic data – such as first name, date and place of birth, field of study, place of domicile, living situation and hobbies – obtained prior the main experiment, we constructed personal profiles of the fictive speakers. They either resembled or differed from the participant’s profile. Similarity was created by using the same gender, first name (or similar equivalents, e.g., *Anna* and *Anne*), same or similar dates and places of birth, same or similar study program, and same hobbies. Dissimilar characters were characterized by not being a student, being around 10 years older, not sharing the birth month and date, living in a different federal state of Germany, having a dissimilar first name, and being interested in different hobbies. Manipulations for every participant were done using the same scheme. The manipulation resulted in four personal profiles of (fictive) speaker characters that resembled the respective participant in her data, and four profiles that differed from the participant’s profile. To detract participants from the study aim, we included trait memory tasks between acquiring the biographical information and the main experiment. Additionally, we instructed the participants to carefully read every profile that was presented during the experiment, as they later should respond to questions regarding bibliographic information.

### Procedure

First, participants filled out questionnaires regarding their demography and their handedness (Oldfield, 1971). After completing the questionnaires, participants were asked to wash their hands and to remove eye make-up. Participants were then seated in a chin rest 72 cm in front of a computer screen. Peripheral physiological measures were recorded from their non-dominant hand, while their dominant hand was free to use a button box for responding. Stimuli were presented via headphones (Sennheiser, HD 449) at a volume of around 55 db. During and shortly after auditory presentation, participants were instructed to fixate a green circle displayed at the center of a screen in order to prevent excessive eye movements. The circle spanned a visual angle of 2.4° × 2.7° and was displayed on an equiluminant gray background. Additionally, they were asked not to move and to avoid blinks during the presentation of target sentences.

**FIGURE 1:**
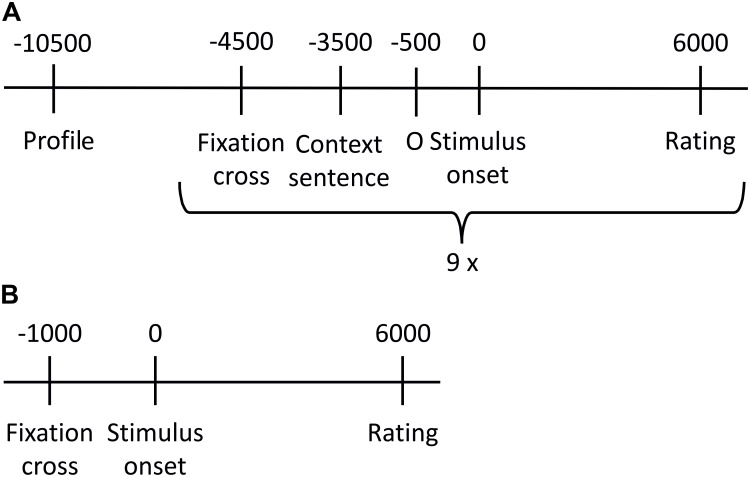
Overview of stimulus presentation procedure. **(A)** One of the 20 presentation blocks created for the prosodic stimuli. All nine stimuli of one block were spoken by the same speaker, and included in randomized order three neutral, three anger and three joy sentences. **(B)** Stimulus presentation of sounds.

The experiment consisted of two parts. **Figure 1** gives an overview about the procedure of the stimulus presentation. Within the first part, prosodic stimuli were presented. Stimuli were presented twice (once in the similar/once in the dissimilar condition), resulting in a total number of 180 stimuli. The stimulus set was divided into 20 blocks of 9 stimuli (three stimuli per emotion category that is anger, neutral, joy). All stimuli within one block were spoken by the same speaker and were presented in random order within a given block. Prior to every prosodic stimulus, a context sentence was presented for 3 s. The personal profile, which manipulated the similarity, was shown prior to each block for 6 s. Every second block was followed by a break. Rating was done 6 s after stimulus onset. Participants had to indicate the valence of each stimulus (positive, negative, and neutral) by pressing one of three buttons. In order to avoid early moving and thus assuring reliable SCR measures, the rating options appeared not until 6 s after stimulus onset and valence-button assignment changed randomly for every trial. Participants were instructed to carefully read the personal profiles and to feel into the speaker and the situation, respectively. This part lasted for about 40 min. At the end of this part, participants answered seven questions regarding bibliographic information of the fictitious speakers.

After a short break, the second part started, in which the 45 emotional sounds were presented. Every trial started with a fixation cross in the middle of the screen for 1 s. The sound was then replayed for 6 s each, while a circle was displayed on screen. When the sound finished, response labels (positive, negative, and neutral) were aligned in a horizontal row on the screen below the circle. The spatial arrangement of the response options was randomly changed for every trial; thus, button order was not predictable. The 45 emotional sounds were presented twice in two independent cycles, each time in randomized order. In analogy to the prosodic part, participants were instructed to listen carefully and to indicate the valence they intuitively associate most with the sounds without elaborative analysis of the sound’s specific meaning. Short breaks were included after every 15th trial. This part of the experiment lasted for about 20 min. The experiment took approximately 60 min in total.

### Psychophysiological Data Recording, Pre-processing, and Analysis

#### Pupil Diameter

Pupil diameter was recorded from the dominant eye using the EyeLink 1000 (SR Research Ltd.), at a sampling rate of 250 Hz. The head position was stabilized via a chin and forehead rest that was secured on the table. Prior to the experiment, the eyetracker was calibrated with a 5-point calibration, ensuring correct tracking of the participant’s pupil. Offline, blinks and artifacts were corrected using spline interpolation. Data was then segmented around stimulus onset (time window: -1000 ms to 7000 ms) and referred to a baseline 500 ms prior stimulus onset. Data were analyzed in consecutive time segments of 1 s duration each. We started the analysis 500 ms after stimulus onset, to allow a short orientation phase, and ended 5500 ms afterward.

#### Skin Conductance

Skin conductance was recorded at a sampling rate of 128 Hz using ActivView and the BioSemi AD-Box Two (BioSemi B.V.). The two Ag/AgCl electrodes were filled with skin conductance electrode paste (TD-246 MedCaT supplies) and were placed on the palm of the non-dominant hand approximately 2 cm apart, while two additional electrodes on the back of the hand served as reference. Offline, data was analyzed using the matlab based software LedaLab V3.4.5 (Benedek and Kaernbach, 2010a). Data was down-sampled to 16 Hz and analyzed via Continuous Decomposition Analysis (Benedek and Kaernbach, 2010a). Skin conductance (SC) is a slow reacting measure based on the alterations of electrical properties of skin after sweat secretion. SC has long recovery times leading to overlapping peaks in the SC signal when SCR are elicited in quick succession. Conducting standard peak amplitude measures is thus problematic, as peaks are difficult to differentiate and subsequent peaks are often underestimated. Benedek and Kaernbach (2010a) developed a method that separates the underlying driver information, reflecting the sudomotor nerve activity (and thus the actual sympathetic activity) from the curve of physical response behavior (sweat secretion causing slow changes in skin conductivity) via standard deconvolution. Additionally, tonic and phasic SC components are separated, to allow a focus on the phasic, event-related activity only. The phasic driver subtracted by the tonic driver is characterized by a baseline of zero. Event-related activation was exported for a response window of 1–6 s after stimulus onset, taking into account the slow signal (Benedek and Kaernbach, 2010b). Only activation stronger than 0.01 μS was regarded as an event-related response (Bach et al., 2009; Benedek and Kaernbach, 2010a). We used averaged phasic driver within the respective time window as measure for SCR. The inter stimulus interval was 2 s for sounds, as rating normally takes around 1 s; 7 s for prosodic stimuli (cf. Recio et al., 2009).

### Reaction Time Task

A subset of participants (20 out of 28, aged 21–30 years, *M* = 24.45) was selected to participate in an additional reaction time task in order to collect behavioral speed and confidence measures of emotion recognition to additionally estimate for potential cognitive difficulties in recognizing the emotional content of stimuli. These measures could not be obtained during the main experiment due to the physiological recordings that were accessed from the non-dominant hand and due to the pupillary recordings that forbid blinks during the critical time window. This part of the study was conducted with a delay of 6 months after the main experiment to ensure that participants did not remember their previous classifications of the stimulus materials. Participants sat in front of a computer screen, and listened to the acoustic stimuli via headphones. They were first confronted with the emotional sounds (first part) in a randomized order and were instructed to stop the stimulus directly as fast as they had recognized the emotion within a critical time window of 6 s. The time window was in accordance to the one in main experiment and corresponded to the durations of sounds. After participants pushed a button, reflecting the time needed for successful emotion recognition, they had to indicate which emotion they perceived (positive, negative, and neutral) and how confident they were in their recognition (likert-scale 1–10), both by paper-pencil. The next trial started after a button press. In the second part, they listened to the prosodic stimuli that had to be classified as expressing joy, anger, or neutral, respectively, within the same procedure as in the first part. The critical time window was again 6 s after stimulus onset.

### Statistical Analysis

Statistical analyses were done in R (R Developmental Core Team, 2012). The similarity manipulation was included into the statistics to account for potential effects of this manipulation. Additionally, this could be seen as a manipulation check. To test the effects of emotion category and similarity on recognition accuracy we built a generalized linear mixed model with binomial error structure (GLMM, lmer function, R package lme4, Bates et al., 2011). Effects on SCRs and pupil size were analyzed using linear mixed models (LMM, lmer function). Models included emotion category, similarity, and the interaction between these two as fixed factors and participant-ID as random factor, to control for individual differences. All models were compared to the respective null model including the random effects only by likelihood ratio tests (function anova). Additionally, we tested the interaction between emotion category and similarity by comparing the full model including the interaction with the reduced model excluding the interaction. We used the model without interaction when appropriate. Models for the emotional sounds included only emotion category as fixed factor and participant-ID as random effect. The models were compared to the respective null models by likelihood ratio tests. Normal distribution and homogeneity of variance for all models were tested by inspecting Quartile–Quartile-Plots (QQ-plots) and residual plots. SCR data deviated from normal distribution and were log transformed. Pairwise *post hoc* tests were conducted using the glht function of the multcomp package (Hothorn et al., 2008) with Bonferroni correction.

In the reaction time task, we did not compare prosody and sounds statistically, knowing about the differences in stimulus length, quantity of stimuli and, regarding the broader perspective, the stimulus structure overall (Bayer and Schacht, 2014). Reaction time data was not normally distributed and was thus log transformed prior to the analysis. Recognition accuracy and reaction time data were only calculated for those stimuli that were responded to within the time window of 6 s, whereas certainty ratings were analyzed for all stimuli in order to not overestimate the ratings. We tested the effect of emotion category on recognition accuracy (using GLMM), reaction time (using a LMM), and certainty ratings (using a cumulative link mixed model for ordinal data, package ordinal, Christensen, 2012) for both prosodic stimuli and emotional sounds. The models include emotion category as fixed factor and participant-ID as random effect. The models were compared to the respective null models by likelihood ratio tests. Pairwise *post hoc* tests were conducted using the glht function with Bonferroni correction for recognition accuracy and reaction time. As cumulative link models cannot be used in the glht *post hoc* tests, we used the single comparisons of the model summary, and conducted the Bonferroni correction separately.

In addition to analyzing the emotion recognition rates in the main experiment and the reaction time task, we also calculated the unbiased hit rates (Hu scores, Wagner, 1993). Recognition rates mirror the listener’s behavior in the actual task, but might be affected by the participant’s bias to preferentially choose one response category. Unbiased hit rates account for the ability of a listener to distinguish the categories by correcting for a potential bias (Wagner, 1993; cf. Rigoulot et al., 2013; Jürgens et al., 2015). We descriptively report the Hu scores in order to provide a complete description of the recognition data, but focused the further analyses on recognition rates only.

## Results

### Emotion Recognition Main Experiment

#### Spoken Utterances With Emotional Prosody

**FIGURE 2:**
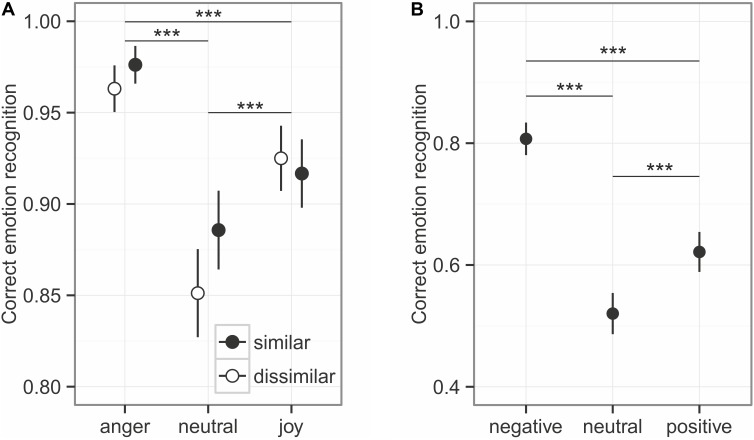
Emotion recognition for prosody **(A)** and sounds **(B)**. Given are the mean values ± 95% CI. Asterisks mark the significance level: ^∗^*p* < 0.05, ^∗∗^*p* < 0.01, ^∗∗∗^*p* < 0.001.

**FIGURE 3:**
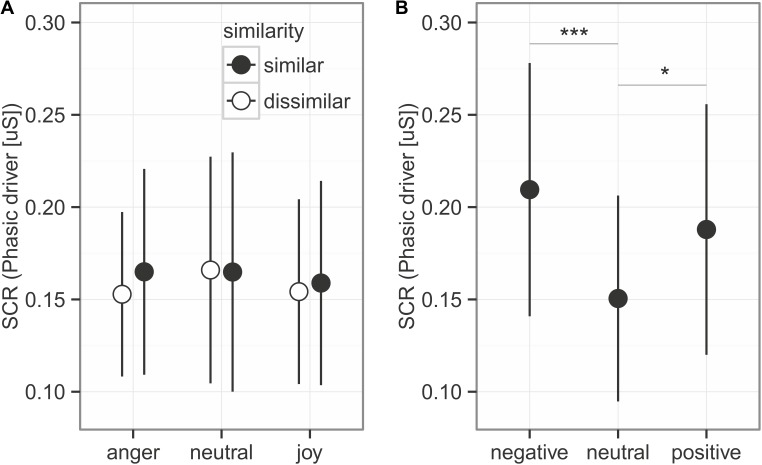
Skin conductance response for the prosodic stimuli **(A)** and the sounds **(B)**. Given is the mean ± 95% CI phasic driver activity within the response window of 1–6 s after stimulus onset. Asterisks mark the significance levels of the *post hoc* tests ^∗^*p* < 0.05, ^∗∗^*p* < 0.01, ^∗∗∗^*p* < 0.001.

Overall, emotional prosody was recognized relatively well, at around 92% (**Figure 2**). The comparison to the null model established an overall effect of the predictors on emotion recognition (χ^2^ = 138.44, *df* = 5, *p* < 0.001), while the interaction between similarity and emotion category was not significant (χ^2^ = 4.53, *df* = 2, *p* = 0.104). Similarity influenced the emotion recognition only at trend level (χ^2^ = 3.21, *df* = 1, *p* = 0.073, **Figure 3**), while emotion category had a strong influence on recognition (χ^2^ = 130.81, *df* = 2, *p* < 0.001). *Post hoc* tests revealed differences in every pairwise comparison (anger vs. joy: *z* = 6.117, *p* < 0.001; anger vs. neutral: *z* = 10.176, *p* < 0.001; joy vs. neutral *z* = 5.088, *p* < 0.001). As can be seen in **Figure 2A**, angry prosody was recognized best, followed by joyful and neutral prosody.

The unbiased hit rates demonstrated that listeners had a generally high recognition ability: Hu_anger_: 0.872 ± 0.122; Hu_neutral_: 0.810 ± 0.190, Hu_joy_: 0.896 ± 0.099 (Mean ± SD). Interestingly, anger does not stick out here, indicating that the high recognition rates of anger might be influenced by a slight bias to rather choose anger as a response, independent of the true emotion category.

#### Affective Sounds

The emotional content of sounds was less accurately recognized than emotional prosody of spoken utterances, with an overall recognition accuracy of around 65% (see **Figure 2B**). Emotion had a significant influence on the recognition, as indicated by the comparison of full model and null model: χ^2^ = 167.52, *df* = 2, *p* < 0.001. With a recognition accuracy of about 52%, neutral sounds had the worst recognition accuracy (negative vs. neutral: *z* = 12.972, *p* < 0.001; negative vs. positive: *z* = 8.397, *p* < 0.001; positive – neutral: 4.575, *p* < 0.001). The Hu scores revealed a low ability of the participants to distinguish the emotion categories: Hu_negative_: 0.554 ± 0.147, Hu_neutral_: 0.323 ± 0.146, Hu_positive_: 0.453 ± 0.166 (Mean ± SD).

### Skin Conductance

#### Spoken Utterances With Emotional Prosody

Skin conductance response (**Figure 3**) represented by the phasic driver activity was not affected by any of the predictors (comparison to null model χ^2^ = 1.605, *df* = 5, *p* = 0.9). This part of the experiment took 40 min. To control whether participants habituated in their response to the emotions due to the long presentation time, we also analyzed only the first half of the experiment, which led to similar results (comparison to null model χ^2^ = 4.910, *df* = 5, *p* = 0.43).

#### Affective Sounds

**FIGURE 4:**
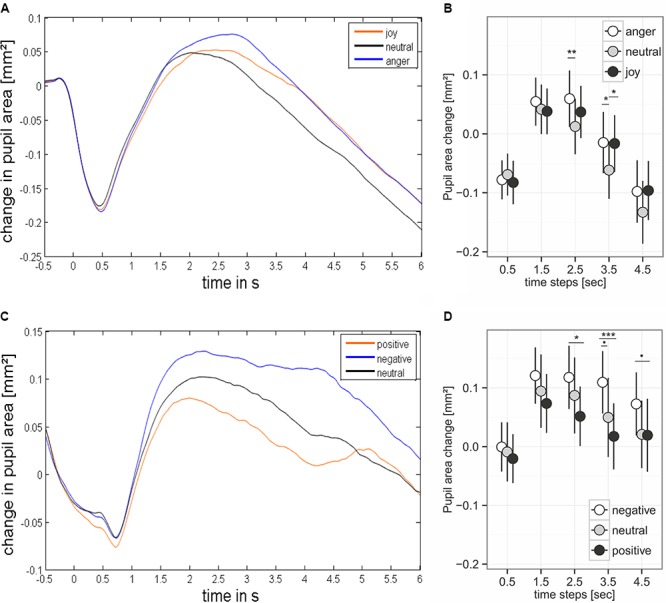
Pupil dilation during presentation of prosodic stimuli **(A,B)** and sound **(C,D)**. **(B,D)** Base on mean values ± 95% CI for the analyzed time steps. Stimulus onset happened at time point 0. Asterisks mark the significance levels of the *post hoc* tests: ^.^*p* < 0.1, ^∗^*p* < 0.05, ^∗∗^*p* < 0.01, ^∗∗∗^*p* < 0.001.

We did find an effect of the emotional sounds on SCR (**Figure 4**; comparison to null model χ^2^ = 15.828, *df* = 2, *p* < 0.001). Consistent with the prediction, more arousing sounds elicited stronger SCRs than neutral sounds (**Figure 3**, *post hoc* tests negative vs. neutral: estimates on log transformed data = 0.336 ± 0.081, *z* = 4.129, *p* < 0.001; positive vs. neutral: estimates on log data = 0.222 ± 0.081, *z* = 2.723, *p* = 0.019). Negative and positive sound elicited SCRs of similar size (negative vs. positive: estimates on log data = 0.114 ± 0.0814, *z* = 1.406, *p* = 0.479).

### Pupil Dilation

#### Spoken Utterances With Emotional Prosody

We found an effect of the predictors on pupil size for the time windows 2.5 – 3.5 and 3.5 – 4.5 seconds after stimulus onset (comparisons to null models, see **Table 3**). There was no interaction between emotion category and similarity on pupil size (**Table 3**). Pupil size was affected by emotion category of speech samples (**Figure 4** and **Table 3**). Interestingly, increases of pupil size dynamically differed between prosodic conditions: Pupil size increased fastly in response to angry stimuli, while responses to joyful stimuli were delayed by about one second (see **Figure 4** and **Table 4**). Neutral stimuli triggered the weakest pupil response in comparison to anger and joy (**Figure 4** and **Table 4**). The similarity condition had no effect on pupil size for the respective time windows (model comparisons χ^2^ < 1.16, df = 1, *p* > 0.28).

**Table 3:** Effects on pupil size for prosody and sounds.

		Null model comparsion^a^			Interaction			Emotion		
Stimulus	Time steps	χ^2^	df	*p*	χ^2^	df	*p*	χ^2^	df	*p*
Prosody	0.5–1.5	9.86	5	0.079.		2				
	1.5–2.5	5.56	5	0.351						
	2.5–3.5	11.56	5	0.041^∗^	0.786	2	0.675	10.66	2	0.005^∗∗^
	3.5–4.5	11.82	5	0.037^∗^	1.63	2	0.444	9.108	2	0.011^∗^
	4.5–5.5	7.722	5	0.172						
Sound	0.5–1.5	1.74	2	0.419						
	1.5–2.5	4.29	2	0.117						
	2.5–3.5	6.95	2	0.031^∗^						
	3.5–4.5	12.81	2	0.002^∗∗^						
	4.5–5.5	6.07	2	0.048^∗^						

^a^*For the sounds, the emotion effect is reflected by the null model comparison. Presented are the results of the model comparison for each time segments. Asterisks mark the significance level: ^∗^*p* < 0.05, ^∗∗^*p* < 0.01.*

**Table 4:** Emotion effects on pupil size for prosody and sounds.

Stimulus	Time step	Emotion		Estimates	*z*-value	*p^a^*
Prosody	2.5–3.5	Joy	Neutral	0.025 ± 0.0143	1.72	0.258
		Anger	Joy	0.023 ± 0.0143	1.58	0.345
		Anger	Neutral	0.047 ± 0.143	3.29	0.003**
	3.5–4.5	Joy	Neutral	0.041 ± 0.017	2.58	0.03*
		Anger	Joy	0.001 ± 0.017	0.1	1
		Anger	Neutral	0.047 ± 0.017	2.67	0.023*
Sounds	2.5–3.5	Positive	Neutral	0.036 ± 0.025	1.43	0.458
		Negative	Positive	0.067 ± 0.025	2.67	0.023*
		Negative	Neutral	0.031 ± 0.025	1.24	0.646
	3.5–4.5	Positive	Neutral	0.032 ± 0.025	1.29	0.592
		Negative	Positive	0.092 ± 0.025	3.68	<0.001***
		Negative	Neutral	0.06 ± 0.25	2.39	0.051
	4.5–5.5	Positive	Neutral	0.001 ± 0.024	0.06	1
		Negative	Positive	0.053 ± 0.024	2.18	0.087
		Negative	Neutral	0.052 ± 0.024	2.12	0.102

^a^*p-values base on Bonferroni correction within one time window. Presented are the results of the post hoc tests for the respective time segments. Asterisks mark the significance level: ^∗^*p* < 0.05, ^∗∗^*p* < 0.01, ^∗∗∗^*p* < 0.001.*

#### Affective Sounds

The pupil size was affected by emotional content of sounds in three time windows (**Table 3** and **Figure 4**). *Post hoc* tests revealed that negative sounds elicited a stronger pupil size response compared to positive sounds (**Table 4**). Differences between negative and neutral sounds almost reached significance. Our results indicate that pupil dilation does not purely reflect arousal differences.

### Emotion Recognition During Reaction Time Task

#### Spoken Utterances With Emotional Prosody

**FIGURE 5:**
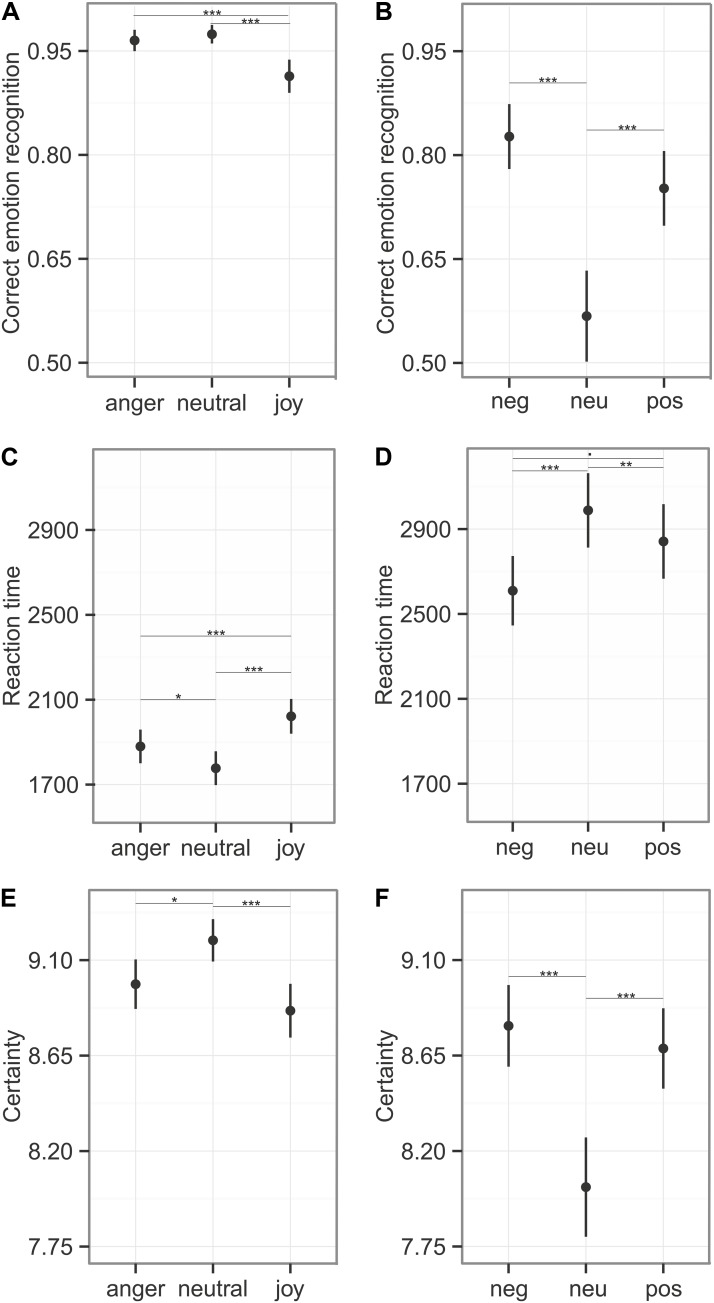
Emotion recognition during Reaction Time Task. The first column **(A,C,E)** depicts the results of the emotional prosody with the emotion categories “anger,” “neutral,” and “joy,” the second column **(B,D,F)** represents the sounds with the categories “negative,” “neutral,”and “positive.” **(A,B)** Correct emotion recognition (mean ± 95% CI) was calculated using stimuli that were responded to within the time window of 6 s. **(C,D)** Reaction time measures (mean ± CI) on stimuli that were responded to within the critical time window. **(E,F)** Certainty ratings (mean ± 95% CI) obtained from the 10 point likert-scale, were calculated for every stimulus. Asterisks mark the significance level: ^.^*p* < 0.1, ^∗^*p* < 0.05, ^∗∗^*p* < 0.01, ^∗∗∗^*p* < 0.001.

Participants responded within the specified time window in 90% of all cases (anger: 91%, neutral: 90%, joy: 89%). We calculated recognition accuracy and reaction times only for these trials. Emotion category had an influence on emotion recognition accuracy (comparison to null model χ^2^ = 26.39, *df* = 2, *p* < 0.001), reaction time (χ^2^ = 42.29, *df* = 2, *p* < 0.001), and the certainty ratings (*LR.stat* = 21.50, *df* = 2, *p* < 0.001). Joy was recognized significantly less accurately (91%) and more slowly (*M* = 2022 ms, see **Figure 5** and **Table 5**). In the certainty ratings, however, judgments for joy did not differ from anger expressions. The unbiased hit rates also demonstrated that the listeners had high recognition ability, indicating that the prosodic utterances could be distinguished easily: Hu_anger_: 0.903 ± 0.112; Hu_neutral_: 0.924 ± 0.120, Hu_joy_: 0.903 ± 0.106 (Mean ± SD). Differences in the recognition rates and the Hu scores of this and the main experiment, might be caused by the fact that only stimuli were entered into this analysis that were responded to within the specified time window.

**Table 5:** *Post hoc* comparisons on emotion recognition, reaction time and confidence ratings for emotional prosody and affective sounds.

Rating	Stimulus	Emotion		Estimates	*z*-value	*p*^a^
Recognition accuracy	Prosody	Neutral	Anger	0.345 ± 0.379	0.909	1
		Joy	Anger	-1.110 ± 0.304	-3.646	<0.001***
		Joy	Neutral	-1.455 ± 0.337	4.321	<0.001***
	Sounds	Neutral	Negative	-1.327 ± 0.217	-6.109	<0.001***
		Positive	Negative	-0.454 ± 0.224	-2.026	0.128
		Positive	Neutral	0.873 ± 0.202	4.315	<0.001***
Reaction time	Prosody	Neutral	Anger	-0.586 ± 0.023	-2.543	0.033*
		Joy	Anger	0.092 ± 0.023	3.976	<0.001***
		Joy	Neutral	0.151 ± 0.023	6.495	<0.001***
	Sounds	Neutral	Negative	0.181 ± 0.403	4.494	<0.001***
		Positive	Negative	0.083 ± 0.039	2.138	0.098
		Positive	Neutral	-0.097 ± 0.040	-2.417	0.047*
Confident ratings	Prosody	Neutral	Anger	0.332 ± 0.118	2.81	0.015*
		Joy	Anger	-0.208 ± 0.116	-1.785	0.223
		Joy	Neutral	-0.540 ± 0.117	-4.608	<0.001***
	Sounds	Neutral	Negative	-0.829 ± 0.148	-5.596	<0.001***
		Positive	Negative	-0.161 ± 0.152	-1.057	0.6
		Positive	Neutral	0.668 ± 0.151	5.413	<0.001***

^a^*Adjusted* p-values (Bonferroni correction). Asterisks mark the significance level: ^∗^*p* < 0.05, ^∗∗∗^*p* < 0.001.

#### Affective Sounds

Participants responded within the specified time window in 81% of all cases (negative: 85%, neutral: 74%, positive: 83%). The recognition varied between emotion categories for recognition accuracy (comparison to null model χ^2^ = 41.41, *df* = 2, *p* < 0.001), reaction time (χ^2^ = 19.97, *df* = 2, *p* < 0.001), and certainty ratings (*LR.stat* = 26.39, *df* = 2, *p* < 0.001). These results indicate difficulties in the categorization of neutral sounds, as reflected in lower accuracy (57% correct), prolonged reaction times (*M* = 2988 ms), and lower certainty ratings (see **Figure 5** and **Table 5**). The Hu scores revealed again a low ability to clearly distinguish between the emotion categories: Hu_negative_: 0.606 ± 0.181, Hu_neutral_: 0.359 ± 0.211, Hu_positive_: 0.551 ± 0.203 (Mean ± SD).

## Discussion

The present study aimed at investigating the elicitation of arousal-related autonomic responses to emotional prosody of spoken utterances in comparison to affective sounds during explicit emotion decisions. As predicted, affective sounds elicited arousal in the perceiver, indicated by increased SCRs to negative and positive sounds as well as enlarged pupil dilations to negative stimuli. Listening to angry and joyful prosodic utterances led to increased pupil dilations but not to amplified SCRs. Biographical similarity between the fictitious speaker and listener employed to increase the social relevance of the spoken stimulus material was ineffective to boost the arousal responses of the listeners.

First of all, our findings indicate that the cues determining emotional prosody in spoken, semantically neutral utterances might be too subtle to trigger physiological arousal to be reflected in changes of electrodermal activity (cf. Levenson, 2014) (see **Figure 3**). These results are in accordance with previous studies on facial expressions that were presented without social context (Alpers et al., 2011; Wangelin et al., 2012). The finding of arousal-related SCRs to affective sounds demonstrated that our participants were generally able to respond sympathetically to auditory stimuli in a lab environment and, importantly, confirmed previous results (Bradley and Lang, 2000).

Emotional prosody differentially affected pupil size reflected in larger dilations for utterances spoken with angry or joyful prosody, which is in line with a study reported by Kuchinke et al. (2011) (see **Figure 4**). Since pupil responses have been demonstrated to reflect the dynamic interplay of emotion and cognition and can thus not only be related to arousal (cf. Bayer et al., 2011), our finding of different effects on pupil size and SCRs is not surprising (see also Urry et al., 2009). Instead, it provides additional evidence that SCRs and pupil responses reflect functionally different emotion- and cognition-related ANS activity. Another previous finding supports the idea that pupil dilation merely reflects cognitive effects on emotional processing. In a study by Partala and Surakka (2003), emotional sounds, taken from the same data base as in our study, triggered stronger pupil dilations for both negative and positive compared to neutral sounds. In our study, however, emotionally negative sounds elicited larger dilations compared to positive sounds, with neutral in between (see **Figure 4**). Procedural differences might explain the inconsistencies between the present and the previous study, as Partala and Surakka did not employ an explicit emotion task. Similar arguments have been made by Stanners et al. (1979), suggesting that changes in pupil size only reflect arousal differences under conditions of minimal cognitive effort. In our study, for both domains – emotional sounds and prosodic utterances – participants had to explicitly categorize the emotional content or prosody of each stimulus. Since accuracy rates provide rather unspecific estimates of cognitive effort, the additional speeded decision task employed in our study, allowed us to analyze the difficulties in recognition of emotional content of both prosody and affective sounds in more detail (see **Figure 5**). Enhanced difficulties in recognizing neutral sounds might explain the unexpected pattern of findings, where neutral sounds elicited larger pupil dilations compared to positive sounds. The detailed analysis of participants’ recognition ability suggests that the recognition of vocally expressed emotions does not require large cognitive resources in general as recognition was quick and accurate. In this case, the impact of emotion on pupil size might be basically caused by arousal (Stanners et al., 1979), even though the arousal level might not have reached a sufficient level to elicit SCRs. While cognitive task effects on SCRs have been demonstrated before (e.g., Recio et al., 2009), we find it unlikely that the SCR modulations in our study reflect cognitive task effects. In our data, the neutral sounds were recognized worst. If the task effects would have affected the SCRs, the responses to neutral sounds should then be increased for neutral sounds compared to affective sounds. The temporal recognition pattern, with neutral classified the most quickly, followed by anger, and joy, classified with the longest delay, fits to the reaction time data found in studies using a gating paradigm (Pell and Kotz, 2011; Rigoulot et al., 2013). The different recognition times might also explain the delay in pupil dilation to joyful prosody. The different recognition times might also explain the delay in pupil dilation to joyful prosody.

Our results raise the question why the processing and classification of affective sounds triggered stronger physiological responses in contrast to emotional prosody (cf. Bradley and Lang, 2000), especially since emotional expressions are presumed to possess a high biological relevance (Okon-Singer et al., 2013). The variation in affective processing of sounds and prosodic utterances might be explained by overall differences between the two stimulus domains. For visual emotional stimuli, Bayer and Schacht (2014) described two levels of fundamental differences between the domains that render a direct comparison almost impossible, namely physical and emotion-specific features. Similar aspects can also be applied to the stimuli used in the present study. Firstly, at the physical level, emotional sounds are more variable in their acoustic content than the spoken utterances. Sounds were hence more diverse, while prosodic emotional expressions vary only in a few acoustic parameters (Hammerschmidt and Jürgens, 2007, see also Jürgens et al., 2011, 2015). Secondly, there are strong differences regarding their emotion-specific features. While pictures and sounds have a rather direct emotional meaning, an emotional expression primarily depicts the expresser’s emotional appraisal of a given situation, rather than the situation itself. Emotional expressions thus possess rather indirect meaning (cf. Walla and Panksepp, 2013). Additionally, our prosodic utterances consisted of semantically neutral sentences. There is evidence that although emotional prosody can be recognized irrespectively of the actual semantic information of the utterance (Pell et al., 2011; Jürgens et al., 2013), semantics seem to outweigh emotional prosodic information when presented simultaneously (Wambacq and Jerger, 2004; Kotz and Paulmann, 2007). Vocal expressions in daily life are rarely expressed without the appropriate linguistic content. Regenbogen et al. (2012), for example, demonstrated that empathic concern is reduced when speech content is neutralized. Prosody is an important channel during emotion communication, but semantics and context might be even more important than the expression alone. Findings might thus be different if the prosodic information and the wording would have been fully consistent. So far, it seems that attending to emotional stimuli such as pictures or sounds seemingly evokes emotional responses in the encoder while attending to emotion expressions in faces or voices rather elicits recognition efforts than autonomic responses (see Britton et al., 2006, for a similar conclusion).

In our study, we aimed at improving the social relevance of speech tokens by embedding them into context and by providing biographical information about the fictive speakers in order to increase the affective reactions of participants toward these stimuli. The lack of effect in our study might indicate that biographical similarity has no effect on emotion processing. It might also be the case that our manipulation was not effective and that similarity unfolds its beneficial effect only in more realistic settings, in which an actual link between both interaction partners can be developed (see Burger et al., 2004; Cwir et al., 2011; Walton et al., 2012). Future research is needed to investigate whether social relevance in more realistic situations, such as avatars looking directly at the participants while speech tokens are presented, or utterances spoken by individually familiar people would increase physiological responsiveness to emotional prosody.

Together, we show that autonomic responses toward emotional prosodic utterances are rather weak, while affective sounds robustly elicit arousal in the listener. Furthermore, our study adds to the existing evidence that pupil size and SCRs reflect functionally different emotion-related ANS activity.

## Author Contributions

RJ, JF, and AS designed the study and wrote the manuscript. RJ conducted the experiments. RJ and AS conducted the data analysis.

## Conflict of Interest Statement

The authors declare that the research was conducted in the absence of any commercial or financial relationships that could be construed as a potential conflict of interest.

## Footnotes

1 Stimulus selection (taken from Bradley and Lang, 1999): Positive sounds: 110, 311, 352, 353, 360, 363, 365, 367, 378, 415, 704, 717, 813, 815, 817 Negative sounds: 134, 261, 281, 282, 289, 380, 423, 501, 626, 699, 709, 711, 712, 719, 910 Neutral sounds: 130, 170, 246, 262, 322, 358, 376, 698, 700, 701, 720, 722, 723, 724, 728
